# Supplementary material for: Superelastic Tellurium Thermoelectric Coatings for Advanced Trimodal Microsensing
Source: Nat Commun. 2026 Jan 13;17:1612. doi: 10.1038/s41467-026-68317-3 (PMC12905337; doi:10.1038/s41467-026-68317-3)
Supplement: Supplementary file 1 — Supplementary Information [file 41467_2026_68317_MOESM1_ESM.pdf]

Supplementary Information for

Superelastic Tellurium Thermoelectric Coatings for Advanced Trimodal Microsensing

Shaowei Cui, Linlin Li, Zi-Xin Huang, Yanzhe Yu, Mingxue Cai, Xiangyin Bao, Chaofan Zhang, Tiandong Zhang, Long Cheng, Wenxuan Zhang, Zheng Lou, Shuo Wang, Wen Gong, Chao-Feng Wu*, Lili Wang*, Yu Wang*

Correspondence to: wuchaofeng@tsinghua-zj.edu.cn; liliwang@semi.ac.cn; yu.wang@ia.ac.cn

This PDF file includes:

Supplementary Note S1. Thermoelectric effect.
Supplementary Note S2. Tactile deep neural networks.
Supplementary Note S3. Visual inpainting deep neural network.
Supplementary Note S4. Thermoelectric effect.
Supplementary Note S5. In-Vivo Experimental Protocol.
Supplementary Fig. S1 | T-scope elastomer property analysis.
Supplementary Fig. S2 | The fabrication process of the T-scope sensor.
Supplementary Fig. S3 | The detailed fabrication of the Te-based thermoelectric subsystem.
Supplementary Fig. S4 | COMSOL simulation of Te-based thermoelectric unit.
Supplementary Fig. S5 | The relationship between output potential and temperature difference.
Supplementary Fig. S6 | Relationship between output potential and contact temperature calibrated using thermocouples.
Supplementary Fig. S7 | The 3D force dataset collection pipeline.
Supplementary Fig. S8 | Overview of the mapping from images to three-dimensional force estimation.
Supplementary Fig. S9 | The quantitative evaluation of the EndoForce network performance on rigid objects.
Supplementary Fig. S10 | An example of the artificial angiography process.
Supplementary Fig. S11 | Visualization of an image restoration example.
Supplementary Fig. S12 | Visualization of an image restoration example.
Supplementary Fig. S13 | In vitro contact experiment of simulated bronchial and stomach model.
Supplementary Fig. S14 | A shot of the bronchus experiment.
Supplementary Fig. S15 | Visual observation restoration effect during endoscopic visual navigation in live animal experiments.
Supplementary Table S1. Network Architecture for Three-dimensional Force Estimation.
Supplementary Table S2. Quantitative results of different inputs.

Supplementary Table S3. Quantitative results of 3D force estimation on different directions.
Supplementary Table S4. Quantitative results of 3D force estimation on different force magnitude.
Supplementary Table S5. PSNR Values (dB) for Different Directions and Sizes.
Supplementary Table S6. Thermal Electric Material Comparison
Supplementary Movie S1. Appearance demonstration of the T-scope sensor.
Supplementary Movie S2. The perceived 3D force vs. ground-truth 3D force testing.
Supplementary Movie S3. Visual-tactile endoscopy experiment of human simulated organ (Bronchus).
Supplementary Movie S4. Visual-tactile endoscopy experiment of human simulated organ (Intestine).
Supplementary Movie S5. Visual-tactile endoscopy experiment of human simulated organ (Stomach).
Supplementary Movie S6. In-vivo animal visual-tactile diagnosis validation experiment.
Supplementary References

Supplementary Notes

Supplementary Note S1. Thermoelectric effect.

To more clearly demonstrate the feasibility of the sensor in a real environment, we employ the COMSOL to simulate the response mechanism of the thermoelectric sensor. The bottom layer of PET is employed to simulate the surface layer of the device being covered by PET, with consideration given to the impact of natural convection on the temperature field distribution. Ecoflex is positioned at the opposite end of the sensor to simulate the actual scenario of the thermoelectric sensor being attached to the top of the visual contact sensor. Subsequently, a contact temperature gradient from 20 °C to 40 °C is established at the contact surfaces to simulate the sensor exposed to different external temperatures. As the contact temperature gradient is modified, the temperature field within the sensor, in addition to the corresponding thermal potential field, exhibits a notable alteration.

With increasing contact temperature, notable differences emerge in the temperature field distributions observed on the Te side and the AuTe alloy side. The AuTe alloy displays a uniform temperature distribution devoid of a considerable temperature gradient, a consequence of its elevated thermal conductivity. As a consequence of its markedly lower lattice thermal conductivity, a considerable decline in the temperature field is observed within the Te polycrystalline film. As the contact temperature rises, the temperature differential between the two ends of the Te side also increases in parallel. At a

contact temperature of 40 °C, a temperature difference of approximately 10 °C is observed between the upper and lower surfaces of Te. In accordance with the Seebeck effect, the potential distribution within the sensor was also elucidated. As the contact temperature increases, it becomes challenging to generate a substantial potential output on the AuTe alloy side, largely due to the uniform temperature distribution across the entire surface. The markedly pronounced temperature disparity between the upper and lower surfaces of Te, conversely, also signifies an evident carrier accumulation effect on a single surface, which may culminate in the genesis of a larger thermal potential. Upon contact at 40 °C, a potential difference of approximately 8 mV is observed between the upper and lower surfaces of the Te side. Furthermore, as the contact temperature rises, the potential output at both ends of the Te film demonstrates a corresponding increase. This phenomenon provides the foundation for the thermoelectric function of the sensor to detect temperature. The thermoelectric properties of the sensor are further investigated.

We first measured the temperature gradient-dependent (ΔT) thermoelectric voltage generation of a single sensor to evaluate the temperature-sensing response of the fabricated device. Considering the difficulty of temperature control and the fact that the expected application is a one-sided stable heat source, we used Peltier heating at the bottom of the device, and the upper part of the device to contact non-flowing air and generate a stable heat field. During the actual measurement, the temperature of the heat source at the bottom of

the device was controlled using the applied voltage of the Peltier, and the actual temperature on both sides of the device was measured using a thermocouple thermometer. The laboratory temperature was maintained constant at 21.4 °C, and the measured output voltage was linearly dependent on the bottom temperature, with a coefficient of 0.323 mV/K. Furthermore, the output voltage was approximately 5.5 mV at a bottom temperature of 39 °C, which is close to body temperature of rabbits, indicating that the device can be used for temperature detection in-vivo. The simulation results shows that the dependence of the measured output voltage on temperature difference from 0 K to 5.3 K, which show good stability of the device. There is a clear linear correlation between the output voltage and temperature difference, and the Seebeck coefficient is approximately 0.647 mV/K, which is significantly higher than that of a Te block.

Supplementary Note S2. Tactile deep neural networks.

The main principle of the T-scope sensor in the endoscopic probe's tip is to convert tactile information into geometric deformations of a silicone layer[1]. It captures various tactile details by imaging the deformations in the geometric shapes that include information about the elastomer's deformation. Specifically, we draw nine densely packed markers between silicone layers. Upon contact, deformation of the gel layer alters the size and position of the attached markers. Given real-time tactile images from the visuotactile sensor of the endoscopic probe, three-dimensional force estimation is approached as a regression problem[2]. The network F_f infers changes in the deformation and size of points in the images, enabling the capture of the entire elastomer's three-dimensional force framework. The force estimation can be detailed as follows:

$$\mathbf{Y}_i = \mathbb{R}_f(\mathbf{X}_i) \subset \mathbb{R}^3$$

Where Y_i denote the three-dimensional force estimation at frame i . X_i indicates the real-time tactile images provided by the visuotactile sensor. The construction of the three-dimensional force estimation network F_f is the central focus of this study's methodology discussion.

The visuotactile sensor's design incorporates nine markers densely distributed on the surface of a silicone elastomer. One marker is positioned at the center, with the remaining eight markers arranged at 45-degree intervals around the center. This configuration allows for accurate capture of deformation information, which is crucial for force estimation.

The core of our network leverages ResNet18 as the backbone for feature extraction. ResNet18, a deep convolutional neural network, is known for its effectiveness in handling image data and its capability to extract hierarchical features through its residual learning framework. The extracted features are then fed into a Multilayer Perceptron (MLP) for final force estimation. The overall structure consists of convolutional layers, batch normalization, ReLU activations, and fully connected layers. The network can be mathematically described as follows:

Given an input image X_i captured by the CMOS camera, the ResNet18 network extracts a feature representation F_r :

$$\mathrm{F}_{r_i} = \mathbb{N}_r(\mathbf{X}_i)$$

This feature representation F_{r_i} is then processed by the MLP to predict the three-dimensional forces Y_i :

$$\mathbf{Y}_i = \mathbb{N}_m(\mathbf{F}_{r_i})$$

Where:

X_i denotes the input image at frame.

i containing deformation information.

F_{r_i} represents the feature vector extracted by ResNet18 for frame i .

Y_i is the output vector representing the estimated three-dimensional forces for frame i .

Specifically: N_r denotes the ResNet18 network, which consists of multiple convolutional layers, batch normalization layers, and ReLU activation

functions, used for extracting features from the input images.

N_m denotes the Multilayer Perceptron (MLP), composed of a series of fully connected layers, batch normalization layers, ReLU activation functions, and dropout layers, used for predicting three-dimensional forces from the extracted features.

The estimated forces Y_i is the output vector representing the estimated three-dimensional forces. consist of the force components along the x, y, and z axes:

$$\mathbf{Y}_i = [F_{x_i} \quad F_{y_i} \quad F_{z_i}]^T$$

To effectively train the network for 3D force estimation, we designed a custom loss function that jointly optimizes both the magnitude and direction of the predicted force vector. The loss function is a weighted composite function that considers the relative error of each force component along the x, y, and z axes, as well as the angular error of the force vector projected onto the YOZ, XOZ, and XOY planes.

We denote the ground-truth 3D force vector by

$$\mathbf{F}_i^{\mathrm{T}} = \left[F_{x_i}^{\mathrm{T}} \quad F_{y_i}^{T} \quad F_{z_i}^{T}\right]^T$$

Where:

$\mathbf{F}_i^{\mathrm{T}}$ represents the true three-dimensional force vector of frame i.

$F_{x_i}^{\mathrm{T}}$ $F_{y_i}^{T}$ $F_{z_i}^{T}$ represents the true strength of x, y and z in the frame i.

To penalize both magnitude and directional deviations, we decompose the objective into two complementary terms:

$$\mathcal{L}_{total} = \mathcal{L}_{mag} + \mathcal{L}_{ang}$$

$\mathcal{L}_{mag}$ is defined as:

$$\mathcal{L}_{mag} = \alpha_x \left| \frac{\sqrt{\sum (F_{x_i})^2} - \sqrt{\sum (F_{x_i}^T)^2}}{\sqrt{\sum (F_{x_i}^T)^2} + \varepsilon} \right| + \alpha_y \left| \frac{\sqrt{\sum (F_{y_i})^2} - \sqrt{\sum (F_{y_i}^T)^2}}{\sqrt{\sum (F_{y_i}^T)^2} + \varepsilon} \right|$$

$$+ \alpha_z \left| \frac{\sqrt{\sum (F_{z_i})^2} - \sqrt{\sum (F_{z_i}^T)^2}}{\sqrt{\sum (F_{z_i}^T)^2} + \varepsilon} \right|$$

α_x, α_y, α_z is the weight coefficient of force loss, which corresponds to the force optimization of x, y and z axes respectively.

$\mathcal{L}_{ang}$ is defined as:

$$\mathcal{L}_{ang} = \beta_x \arctan\left(\frac{F_{x_i} - F_{x_i}^T}{\sqrt{(F_{y_i} - F_{y_i}^T)^2} + \sqrt{(F_{z_i} - F_{z_i}^T)^2} + \varepsilon} \right) + \beta_y \arctan\left(\frac{F_{y_i} - F_{y_i}^T}{\sqrt{(F_{x_i} - F_{x_i}^T)^2} + \sqrt{(F_{z_i} - F_{z_i}^T)^2} + \varepsilon} \right)$$

$$+ \beta_z \arctan\left(\frac{F_{z_i} - F_{z_i}^T}{\sqrt{(F_{x_i} - F_{x_i}^T)^2} + \sqrt{(F_{y_i} - F_{y_i}^T)^2} + \varepsilon} \right)$$

α_x, α_y, α_z is the weight coefficient of angle loss, and the projection angles of stress vectors in YOZ, XOZ and XOY planes are optimized respectively.

$\varepsilon = 10^6$ prevents division by zero and α_x, α_y, α_z, β_x, β_y, β_z follows the following definitions:

$$w = \frac{\alpha_x}{\beta_x} = \frac{\alpha_y}{\beta_y} = \frac{\alpha_z}{\beta_z}$$

The weight-balancing factor w governs the relative importance between force-magnitude optimization and directional regularization:

$w > 1$ emphasizes magnitude accuracy, whereas $w < 1$ prioritizes directional consistency.

$$\alpha_x = \frac{w \cdot r_1}{(r_1 + r_2 + r_3) + (r_1 + r_2 + r_3)}$$

$$\alpha_y = \frac{w \cdot r_2}{(r_1 + r_2 + r_3) + (r_1 + r_2 + r_3)}$$

$$\alpha_z = \frac{w \cdot r_3}{(r_1 + r_2 + r_3) + (r_1 + r_2 + r_3)}$$

$$\beta_x = \frac{r_1}{(r_1 + r_2 + r_3) + (r_1 + r_2 + r_3)}$$

$$\beta_y = \frac{r_2}{(r_1 + r_2 + r_3) + (r_1 + r_2 + r_3)}$$

$$\beta_z = \frac{r_3}{(r_1 + r_2 + r_3) + (r_1 + r_2 + r_3)}$$

r_1, r_2, r_3 is the weight coefficient of angle loss, and the projection angles of stress vectors in YOZ, XOZ and XOY planes are optimized respectively.

The experimental results show that when $w = 1$, $r_1{:}r_2{:}r_3 = 1:1:8$, the performance of the model is the best.

As shown in Supplementary Fig. S9, the mean errors in the X, Y, and Z components were 0.019 N, 0.015 N, and 0.061 N, respectively. Supplementary Table S2 shows that the tactile coating pattern after segmented realize better force perception (RMSE: 0.238 N vs 0.084 N, R^2 : 0.665 vs 0.851).

Supplementary Table S3 presents the RMSE and mean errors in the X, Y, and Z components for different directions. The RMSE values vary across directions, with the lowest RMSE observed for the "down" direction (0.067 N) and the highest for the "center" direction (0.095 N).

Supplementary Table S4 shows the RMSE and mean errors in the X, Y, and Z components for different models categorized by size (Big and Small). The "Big" model exhibits a lower RMSE (0.077 N) compared to the "Small"

model (0.094 N). The mean errors for the "Big" model in the X, Y, and Z components are 0.019 N, 0.014 N, and 0.057 N, respectively, while the "Small" model shows mean errors of 0.011 N, 0.014 N, and 0.069 N. In addition, the two are superficially similar in terms of R^2, indicating that the proposed EndoForce network is sufficiently robust and insensitive to the force magnitude.

These results indicate that the proposed three-dimensional force estimation network can effectively estimate the three-dimensional forces acting on the probe and perform well under various conditions. This highlights the potential of the method for practical applications.

Supplementary Note S3. Visual inpainting deep neural network.

The distinctive marker layer pattern on the T-scope probe, while essential for its tactile sensing capabilities, introduces visual artifacts that can detract from the original visual observation quality of the endoscopy system. These visual disturbances, or "visual pollution," interfere with the clarity and accuracy of the images produced by the endoscope, potentially impacting diagnostic outcomes. Traditional inpainting techniques typically address such issues by filling in the missing or obscured image pixels based on the surrounding features. However, recent advancements in generative image modeling have significantly enhanced the effectiveness of inpainting methods.

Given a masked video sequence $X=\{X_t \in \mathbb{R}^{H\times W\times 3}\}_{t=1}^{T}$, with a temporal length of T, and its corresponding binary mask sequence $M=\{M_t \in \mathbb{R}^{H\times W\times 1}\}_{t=1}^{T}$, the objective of video inpainting is to generate visually consistent and coherent content within the corrupted or missing regions[3,4,5,6]. Before processing the sequence through the proposed framework, forward and backward optical flow sequences, denoted as $F^f=\{F_t^f=F_{t\rightarrow t+1} \in \mathbb{R}^{H\times W\times 2}\}_{t=1}^{T-1}$ and $F^b=\{F_t^b=F_{t+1\rightarrow t} \in \mathbb{R}^{H\times W\times 2}\}_{t=1}^{T-1}$, are estimated from the input video X. These flow sequences are initially incomplete due to the masked regions and are subsequently refined using a Recurrent Flow Completion module to reconstruct accurate flow fields. Guided by the completed flows, a two-stage propagation process is performed: first, global image propagation utilizes flow-guided information across frames to fill

missing regions holistically, followed by local feature propagation that refines details using spatially neighboring features.

The propagated features are then enhanced through multiple Multi-Scale Spatiotemporal Video Transformer blocks to model hierarchical spatiotemporal dependencies. Finally, a decoder reconstructs the refined features into the inpainted video sequence $\hat{Y} = \left\{\hat{Y}_t \in \mathbb{R}^{H \times W \times 3}\right\}_{t=1}^{T}$. The framework integrates flow completion, global-local propagation, multi-scale spatiotemporal refinement, and reconstruction to achieve temporally coherent and visually realistic video inpainting results. In this study, we employ a Denoising Diffusion Probabilistic Model (DDPM) for image inpainting to tackle the visual pollution caused by the T-scope marker patterns. To create a robust inpainting dataset, we first developed a version of the probe without the marker texture layer, allowing us to capture clear tactile images free from visual artifacts. We then artificially introduced noise that mimics the marker pattern into these images, creating paired datasets where the original, uncorrupted image serves as the ground truth. This approach allows us to train the diffusion model effectively, teaching it to reconstruct the obscured areas with high accuracy.

Once trained, the transformer-based network, with its learned weights, can accurately restore the visual information in the regions affected by the marker pattern. This method not only removes unwanted visual artifacts but also preserves the overall image quality, ensuring that the endoscopy system retains

its full diagnostic capability while benefiting from the enhanced tactile sensing provided by the T-scope probe.

Supplementary Note S4. Thermoelectric effect.

Temperature sensors are primarily categorized into non-contact and contact types. Non-contact sensors are susceptible to external interference and significant detection errors due to variations in the emissivity of the target object. Therefore, contact-type temperature sensors are preferred.

Contact-type temperature sensors mainly include thermistors and thermocouples. Thermistors primarily utilize precious metals and semiconductors as sensitive materials, measuring temperature by detecting changes in their internal resistance under thermal influence. These sensors typically exhibit minimal internal resistance changes, resulting in relatively small variations in the generated electrical signal. Furthermore, achieving high-precision signal detection requires complex bridge circuit calibration, which necessitates multiple lead wires—making implementation on flexible micro-probes impractical. Additionally, ensuring spatial measurement accuracy and minimizing external circuit variations demands constructing high-resistance sensitive units within a 1 mm diameter circle, with multiple wires extending outward. This necessitates the introduction of micro/nano fabrication techniques, significantly increasing process complexity. In contrast, thermocouple sensors do not require complex wiring design and can be

structurally customized according to visual and tactile perception requirements to meet our needs.

Theoretical and experimental research on thermocouple sensing materials is relatively mature. Existing research indicates that tellurium and its compounds exhibit outstanding thermoelectric properties (Supplementary Table S6). Notably, recent studies demonstrate that tellurium nanomaterials have extremely low thermal conductivity (facilitating pronounced temperature gradients) and exceptional Seebeck coefficients (yielding higher signal outputs for the same temperature difference), making them highly suitable for thermoelectric sensing unit fabrication. This is the rationale behind selecting Te thin films for device construction and optimization in this work.

Supplementary Note S5. In-Vivo Experimental Protocol.

The experiment uses 6 Changbai Mountain white rabbits, which are randomly divided into 3 groups (n=2 per group): Control group – given by an equal volume of normal saline via gavage; 2.5 ml/kg absolute ethanol group – given by absolute ethanol via gavage at a dose of 2.5 ml per kg of body weight; 5 ml/kg absolute ethanol group – given by absolute ethanol via gavage at a dose of 5 ml per kg of body weight. The rabbits are fasted for 24 hours before the experiment but have free access to drinking water.

For gastric ulcer model establishment, absolute ethanol is administered via a single gavage (at doses of 2.5 ml/kg or 5 ml/kg respectively) to induce acute gastric ulcer models. For gastroscopic observation, 2 hours after ethanol gavage, the rabbits are anesthetized (anesthesia method and dose refer to relevant literature or animal laboratory standards). Subsequently, an animal gastroscope is used to observe and record the gastric mucosa. Key observations focus on gastric mucosal hyperemia, edema, bleeding points, erosion, and ulcer formation. The degree of gastric mucosal injury is evaluated using the Ulcer Index (UI) scoring method, and typical endoscopic images are saved.

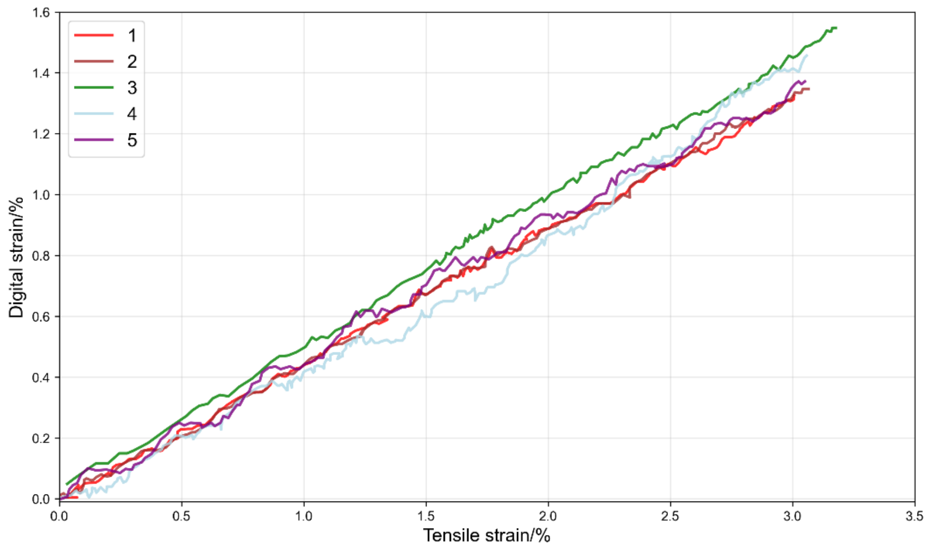

Supplementary Fig. S1 | T-scope elastomer property analysis. This figure shows the relationship between the tensile strain and digital strain of the elastomer used in the T-scope sensor. Source data are provided as a Source Data file.

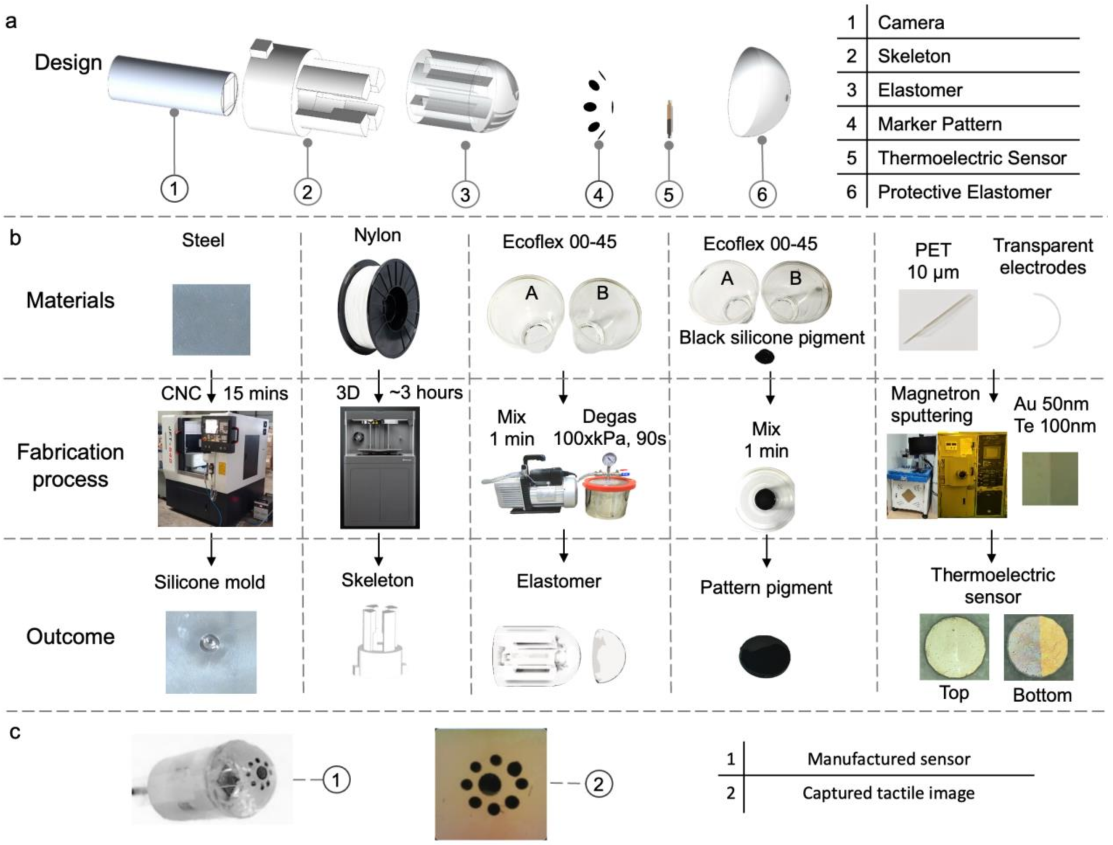

Supplementary Fig. S2 | The fabrication process of the T-scope sensor. **a** An exploded view of the T-scope probe with all parts in the design; bold items were custom-fabricated. **b** The materials, processing steps and

intermediate outcomes for the elastomer, the skeleton frame, the molds for over-molding and the pattern. **c** The partially assembled T-scope and an image captured under contact.

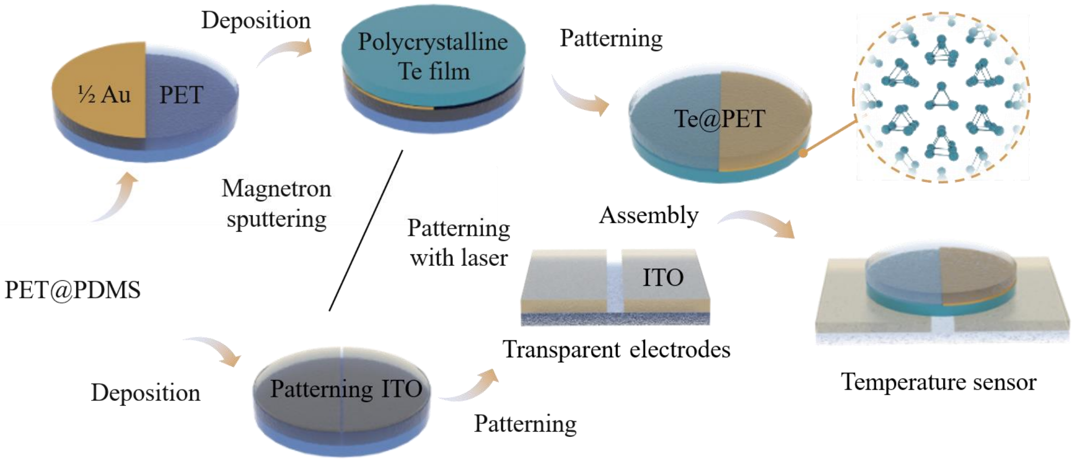

Supplementary Fig. S3 | The detailed fabrication of the Te-based thermoelectric subsystem.

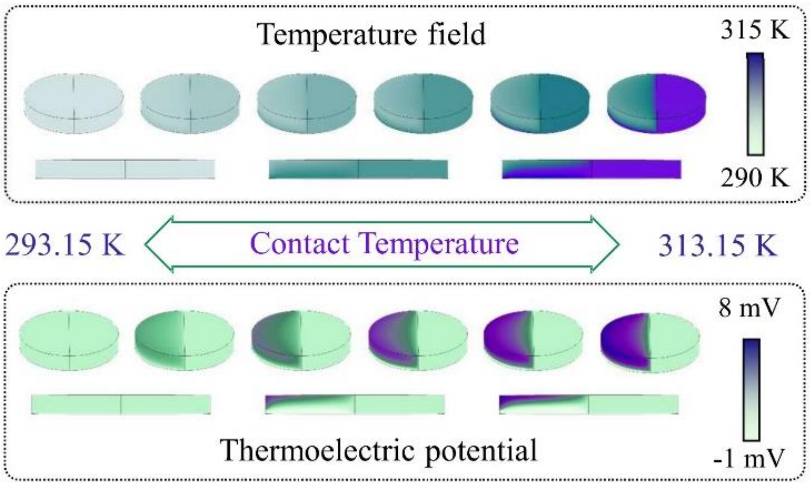

Supplementary Fig. S4 | COMSOL simulation of Te-based thermoelectric unit. Visualization of the temperature field and thermoelectric potential distribution of Te-based thermoelectric unit at contact temperatures ranging from 20 to 40 degrees Celsius.

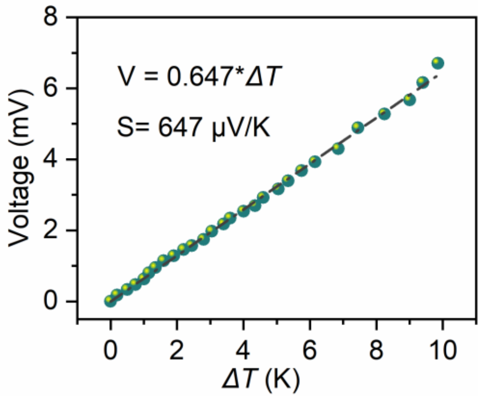

Supplementary Fig. S5 | The relationship between output potential and temperature difference. The output potential of the Te-based thermoelectric unit versus temperature difference. Source data are provided as a Source Data file.

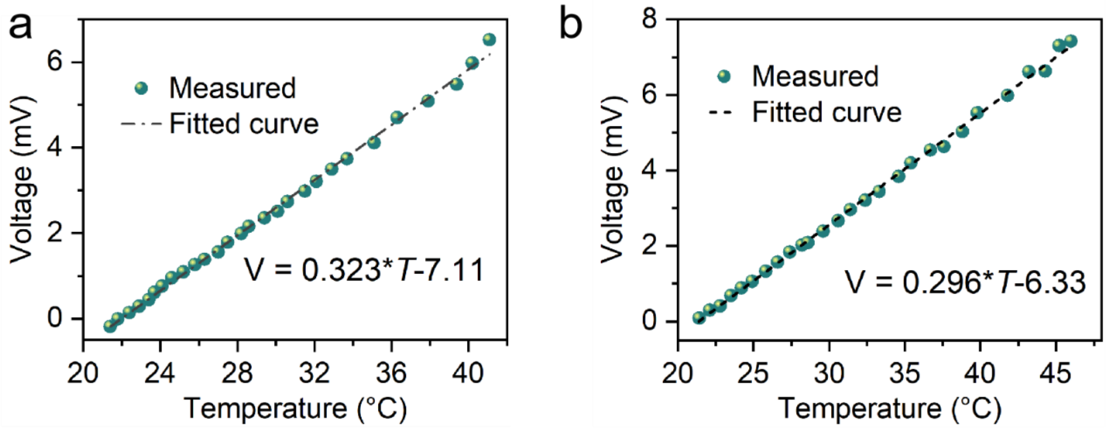

Supplementary Fig. S6 | Relationship between output potential and contact temperature calibrated using thermocouples. a Te-based thermoelectric unit. **b** Te-based thermoelectric subsystem in T-scope. Source data are provided as a Source Data file.

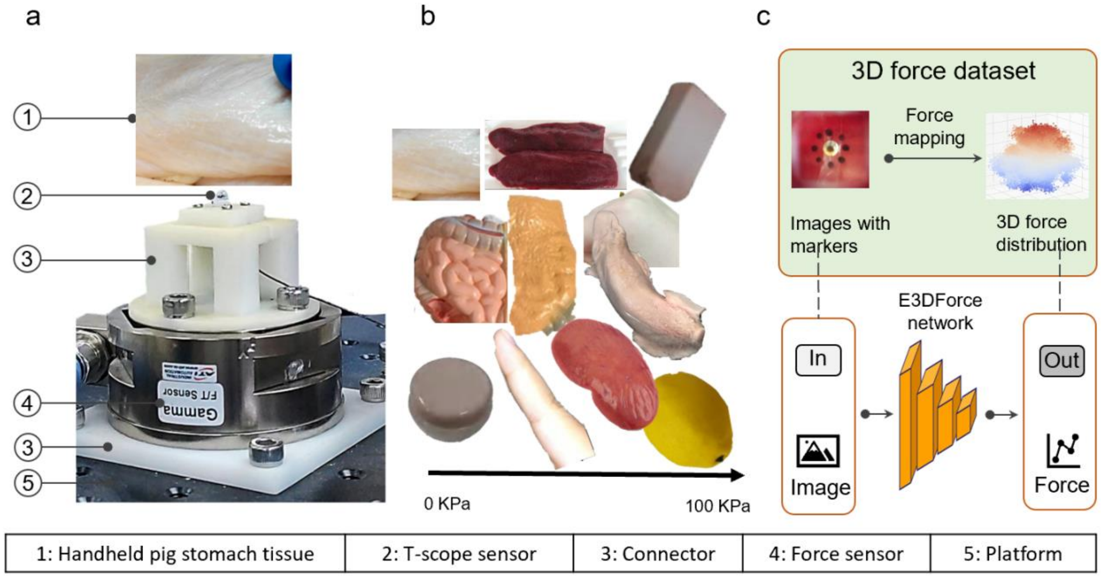

Supplementary Fig. S7 | The 3D force dataset collection pipeline. a The T-scope sensor is fixed on a standard ATI six-axis force/torque sensor, and a real piece of pig stomach tissue is contacted with the sensor surface manually. **b** A dozen objects covering the hardness range of 0-100KPa are used for data collection. **c** The 3D force dataset contains abundant tactile image-force pairs, and each sample provides a ground-truth 3D force label under various contact scenes.

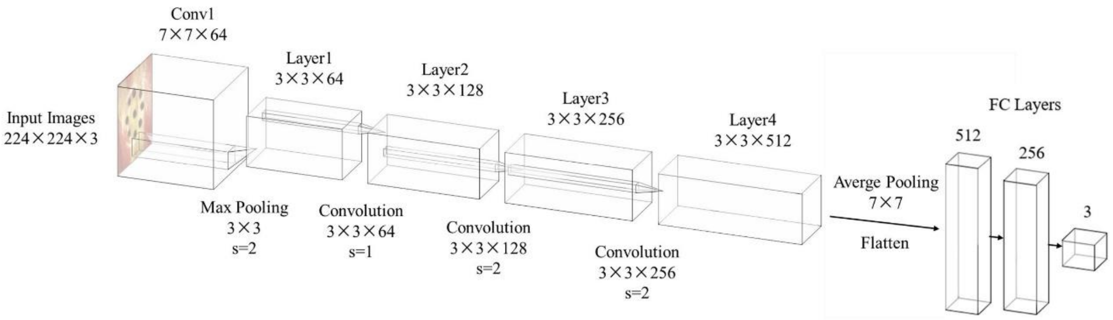

Supplementary Fig. S8 | Overview of the mapping from images to three-dimensional force estimation. In this paper, images are used as input to estimate three-dimensional forces through the EndoForce network.

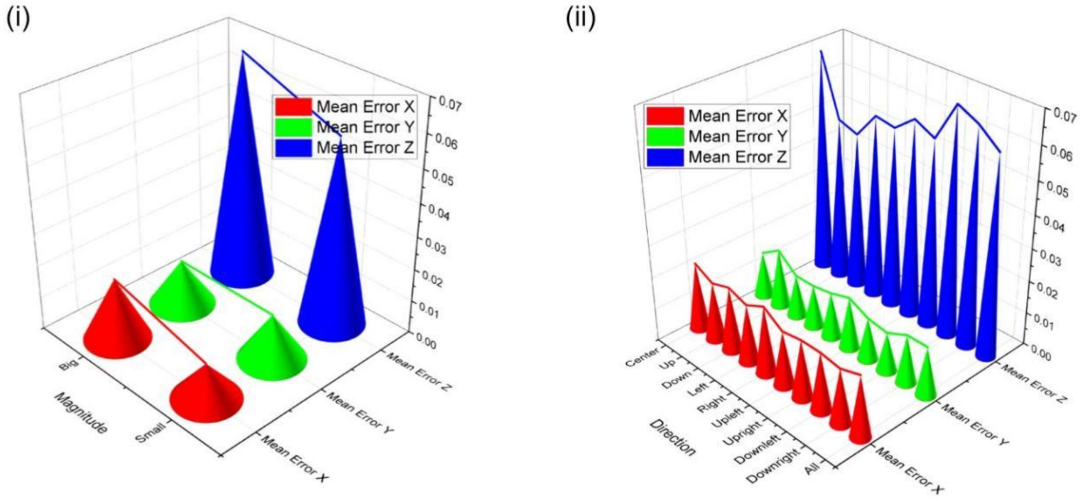

Supplementary Fig. S9 | The quantitative evaluation of the EndoForce network performance on rigid objects. (i) Contact directions. **(ii)** Force amplitude. Source data are provided as a Source Data file.

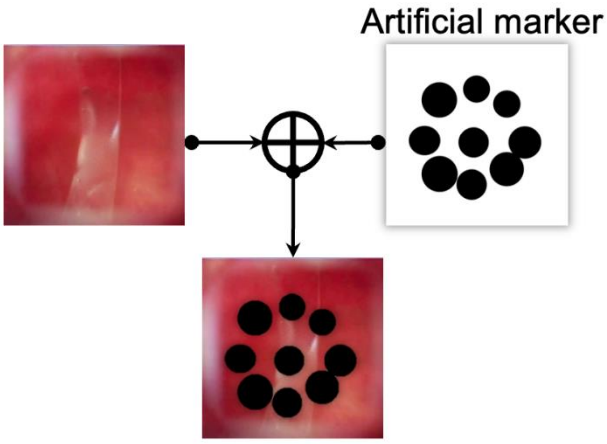

Supplementary Fig. S10 | An example of the artificial angiography process.

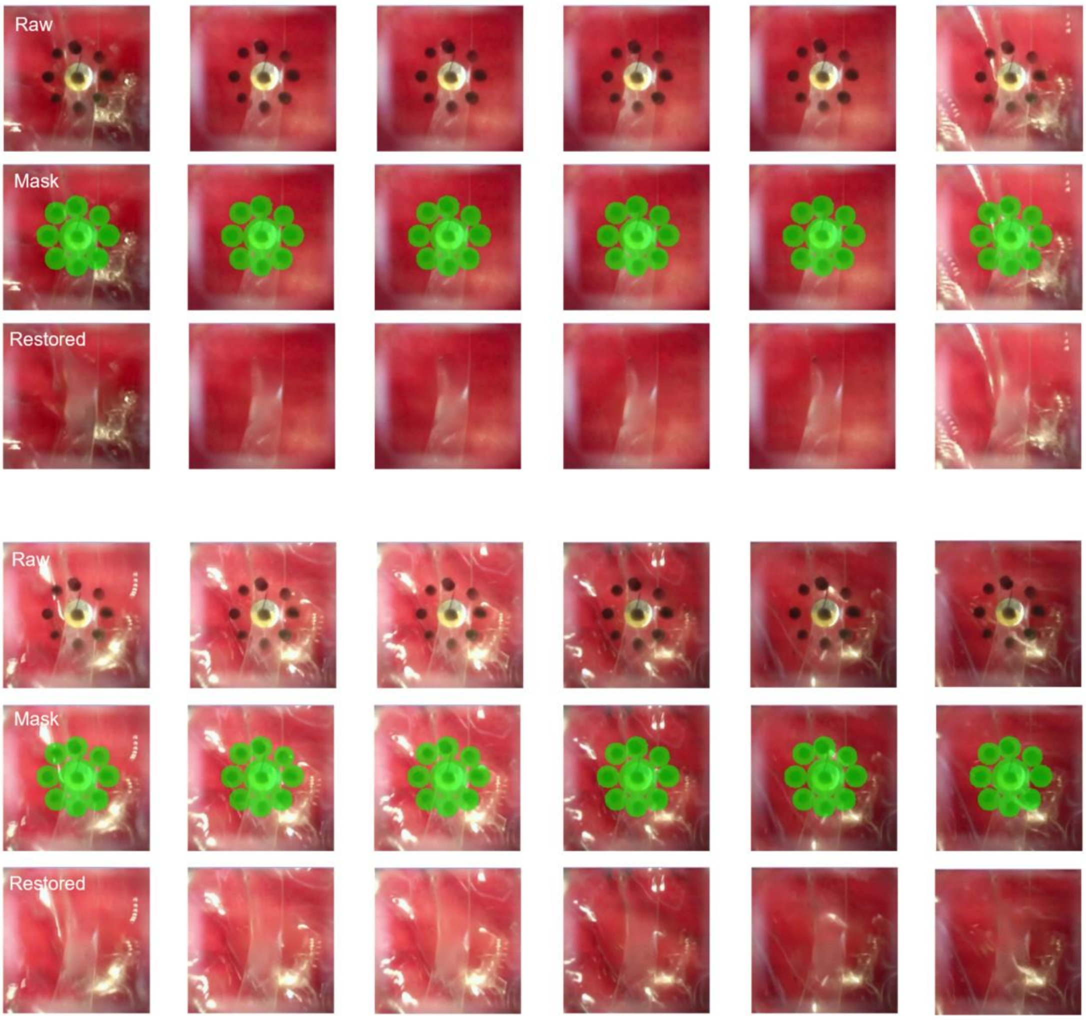

Supplementary Fig. S11 | Visualization of an image restoration example. We place a probe with a dot array imprint on the internal stomach tissue of a dissected rabbit, creating a visual image and the restoration effect.

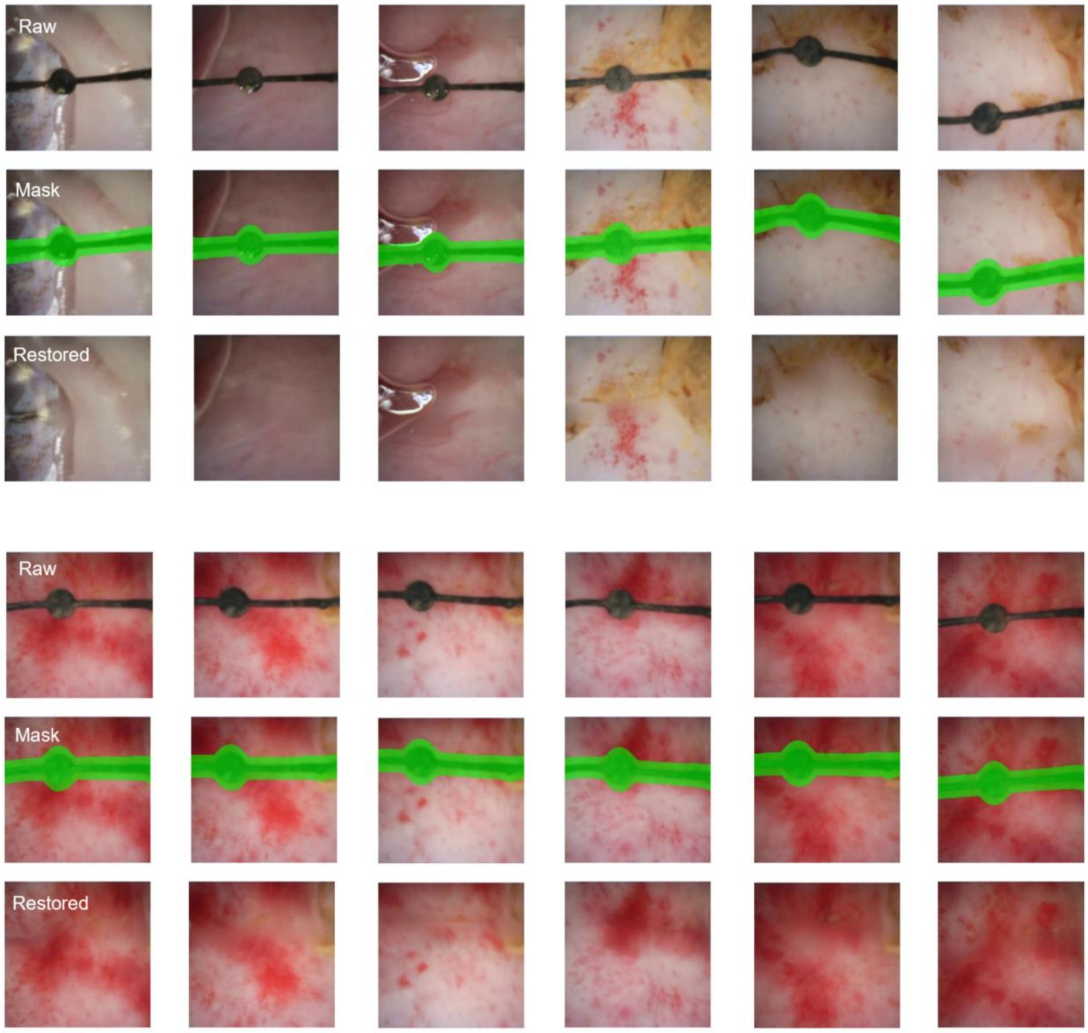

Supplementary Fig. S12 | Visualization of an image restoration example. We place a probe with a dot-line array imprint on the oral cavity, esophagus, and stomach of a living rabbit, generating visual images and the restoration results.

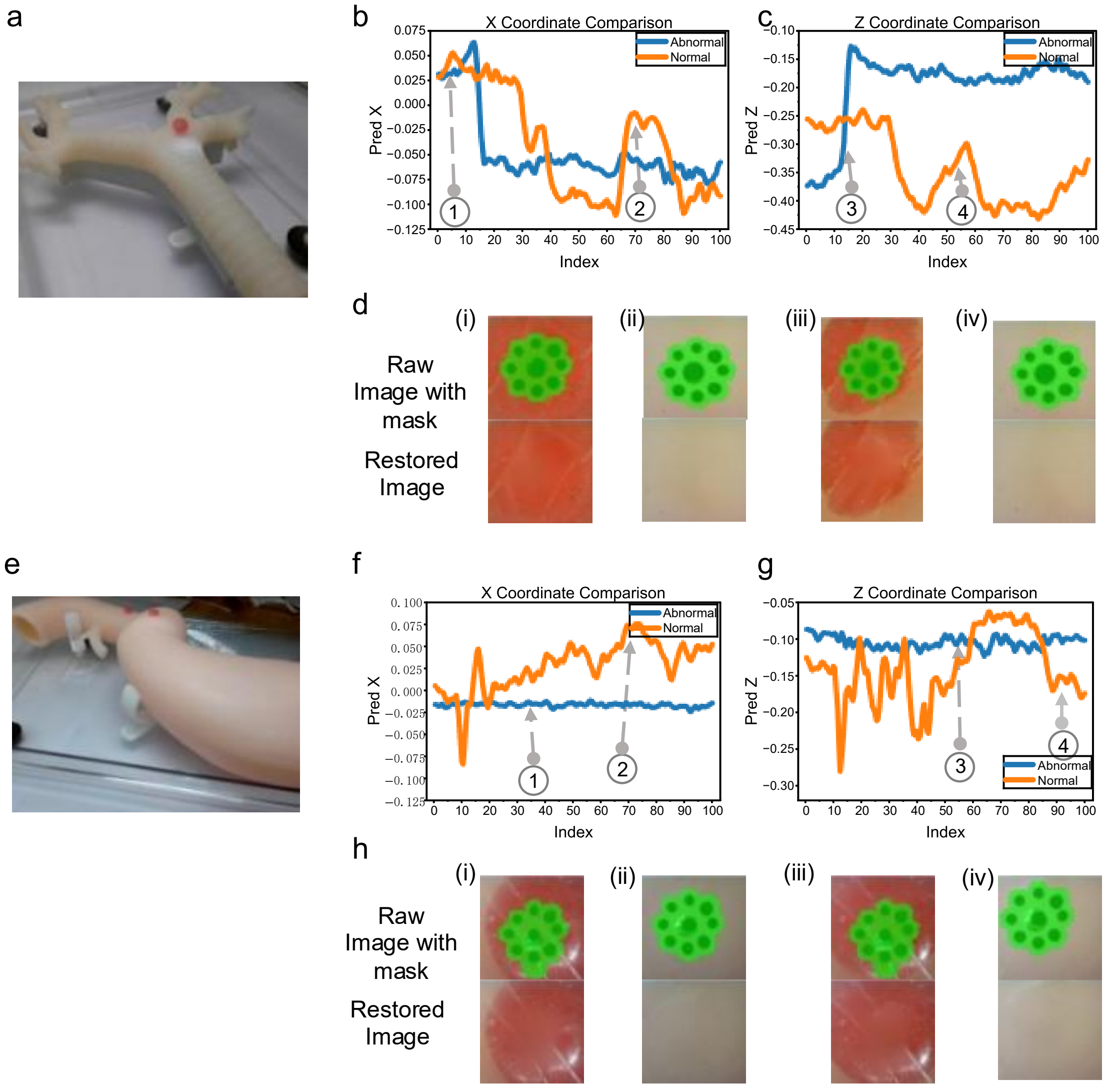

Supplementary Fig. S13 | In vitro contact experiment of simulated bronchial and stomach model. a The operator manually navigates the endoscopic system equipped with the T-scope probe to simulate contact bronchial model interactions. **b** Comparison of contact force estimation curves between normal and abnormal tissues in bronchial model (x-axis). **c** Comparison of contact force estimation curves between normal and abnormal tissues in bronchial model (z-axis). **d** Visualization of image restoration performance across different tissues at various time points in bronchial model. **e** The operator manually navigates the endoscopic system equipped with the T-scope probe to simulate contact stomach model interactions. **f** Comparison of contact force estimation curves between normal and abnormal tissues in stomach model (x-axis). **g** Comparison of contact force estimation curves between normal and abnormal tissues in stomach model (z-axis). **h** Visualization of image restoration performance across different tissues at various time points in stomach model. Source data are provided as a Source Data file.

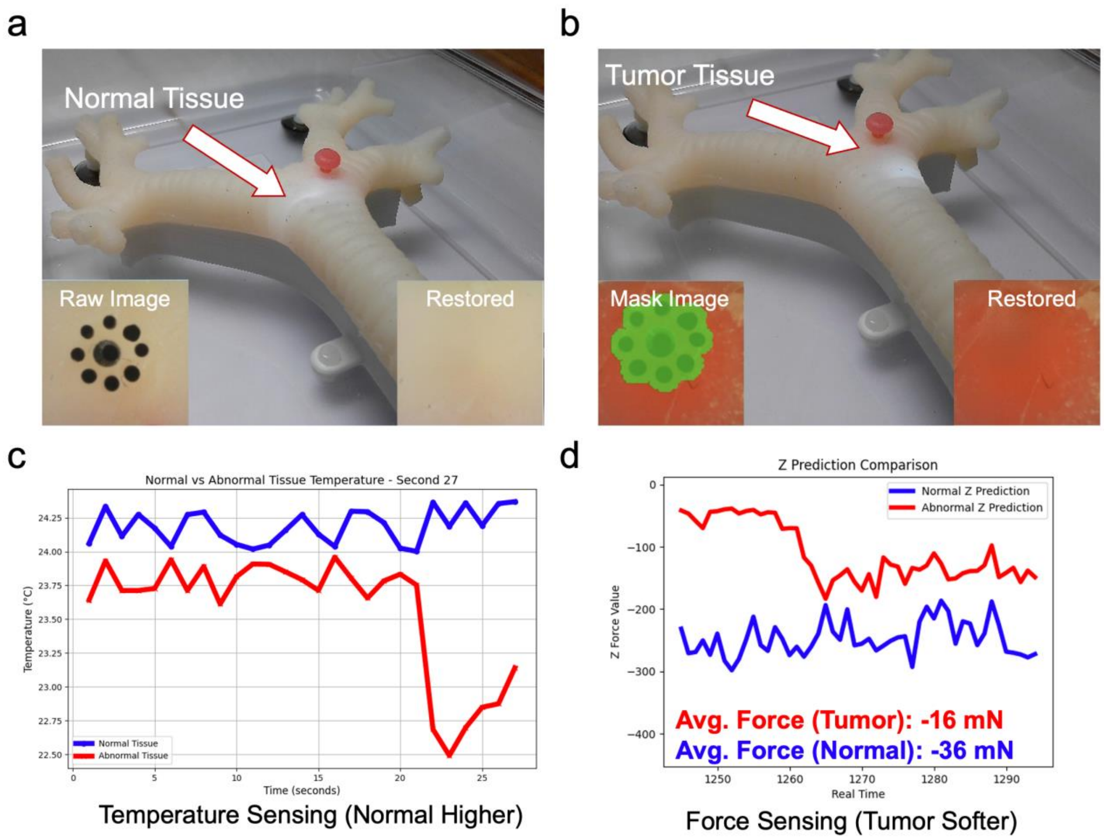

Supplementary Fig. S14 | A shot of the bronchus experiment. a Comparison of visual images and repair effects of normal tissue. **b** Comparison of visual images and repair effects of tumor tissue. **c** The temperature of normal tissue and abnormal tissue. **d** The average force of tumor tissue (-16 mN) is less than that of normal tissue (-36 mN). Source data are provided as a Source Data file.

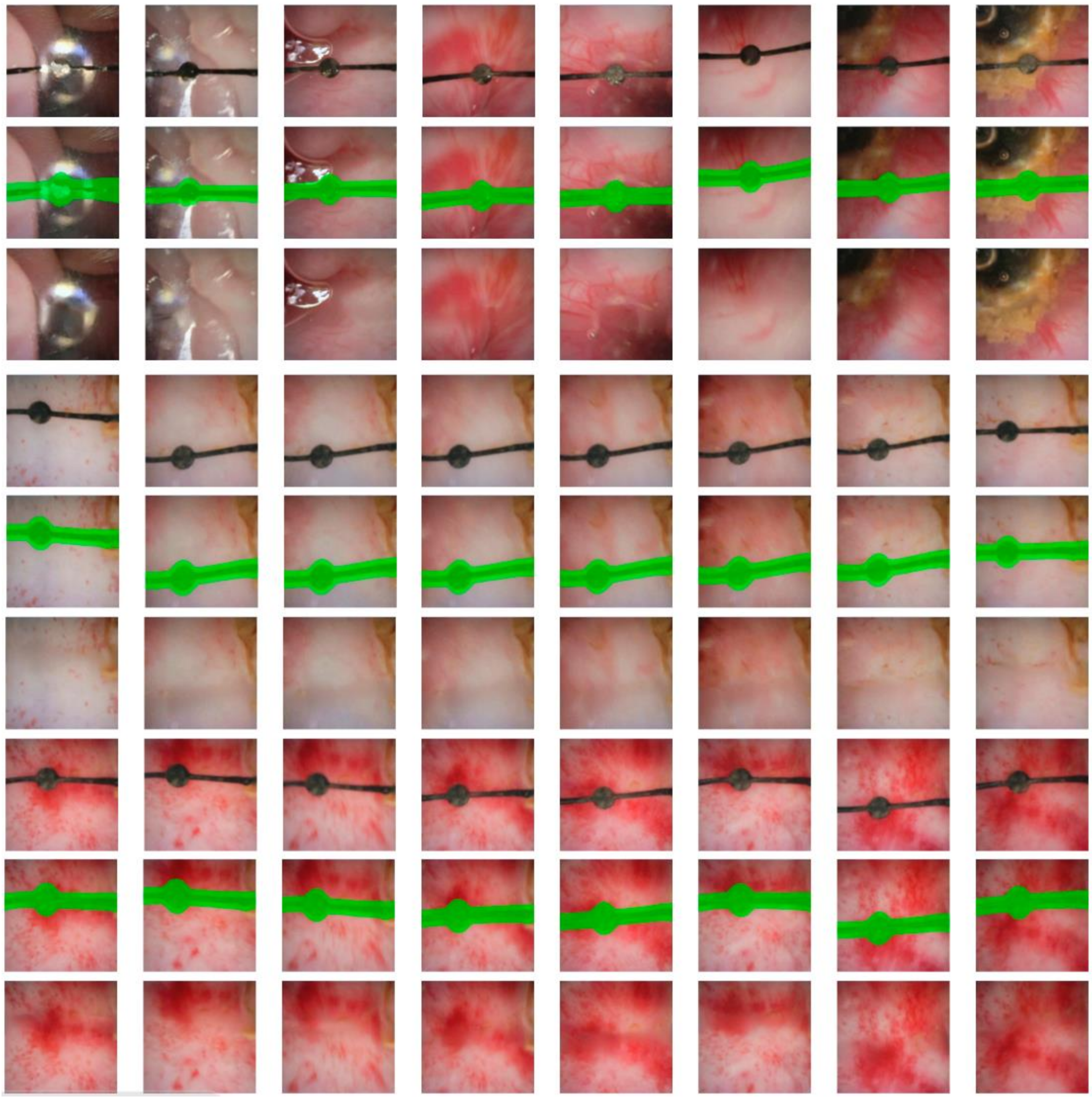

Supplementary Fig. S15 | Visual observation restoration effect during endoscopic visual navigation in live animal experiments.

Supplementary Table S1. Network Architecture for Three-dimensional Force Estimation.

Layer Type	Layer Details	Output Shape
**ResNet18**	Conv1: 7x7, 64, stride 2, padding 3	(64, 112, 112)
	MaxPool: 3x3, stride 2	(64, 56, 56)
	Layer1: 2x(3x3, 64)	(64, 56, 56)
	Layer2: 2x(3x3, 128), stride 2	(128, 28, 28)
	Layer3: 2x(3x3, 256), stride 2	(256, 14, 14)
	Layer4: 2x(3x3, 512), stride 2	(512, 7, 7)
	AvgPool: 7x7	(512, 1, 1)
**MLP**	Linear: 512 → 512	(512)
	Linear: 512 → 256	(256)
	Linear: 256 → 3	(3)

Supplementary Table S2. Quantitative results of different inputs.

Image Type	RMSE				MAE			R^2
	X	Y	Z	Total	X	Y	Z	
**Origin**	0.100	0.134	0.327	0.368	0.076	0.093	0.257	0.665
**Segment Anything**	0.019	0.015	0.061	0.084	0.019	0.015	0.061	0.851

Supplementary Table S3. Quantitative results of 3D force estimation on different directions.

Direction	RMSE				MAE			R^2
	X	Y	Z	Total	X	Y	Z	
**Center**	0.021	0.014	0.068	0.095	0.021	0.014	0.068	0.839
**Down**	0.020	0.013	0.048	0.067	0.020	0.013	0.048	0.812
**Downleft**	0.018	0.012	0.069	0.090	0.018	0.012	0.069	0.526
**Downright**	0.017	0.015	0.066	0.086	0.017	0.015	0.066	0.779
**Left**	0.018	0.013	0.056	0.075	0.081	0.013	0.056	0.718
**Right**	0.021	0.014	0.055	0.077	0.021	0.014	0.055	0.594
**Up**	0.018	0.018	0.050	0.068	0.018	0.018	0.050	0.731
**Upleft**	0.017	0.016	0.060	0.081	0.017	0.016	0.060	0.714
**upright**	0.018	0.013	0.057	0.076	0.018	0.013	0.057	0.872

Supplementary Table S4. Quantitative results of 3D force estimation on different force magnitude.

Magnitude	RMSE				MAE			R^2
	X	Y	Z	Total	X	Y	Z	
**Big**	0.019	0.014	0.057	0.077	0.019	0.014	0.057	0.744
**Small**	0.011	0.014	0.069	0.094	0.011	0.014	0.069	0.852

Supplementary Table S5. PSNR Values (dB) for Different Directions and Sizes.

Direction/Size	up	down	center	left	right	upleft	upright	downleft	downright
**Big(avg)**	36.92	36.42	36.61	36.33	35.90	36.56	36.28	36.37	36.19
**Medium(avg)**	37.69	37.27	37.36	37.04	36.68	37.30	37.05	37.10	37.07
**Small(avg)**	38.38	38.04	38.31	38.00	37.71	38.13	37.91	37.92	37.91
**Big(max)**	37.64	37.56	37.42	37.09	36.68	37.46	37.27	37.56	37.29
**Medium(max)**	38.71	38.59	38.09	37.86	37.45	38.20	37.87	38.18	38.21
**Small(max)**	39.36	39.21	39.20	38.94	38.67	39.44	38.66	38.81	38.80
**Big(min)**	35.91	35.24	35.64	35.33	35.14	35.66	35.57	35.14	34.84
**Medium(min)**	36.62	35.86	36.61	36.13	35.57	36.38	36.14	36.19	36.03
**Small(min)**	37.35	36.65	37.28	36.89	36.42	37.26	37.14	37.06	37.15

Supplementary Table S6. Thermal Electric Material Comparison

Material	Structure (Horizontal/ Vertical)	Sensitivity (μV/K)	Process temperature(℃)	Dimensions	References
BiSbTe	V	200	610	2.6 mm	Ref.7
$Bi_2Te_3/Bi_{0.5}Sb_{1.5}Te_3$	H	354	250	5 μm	Ref.8
p-BiSbTe	V	240	600	10-12 mm	Ref.9
$Bi_{0.5}Sb_{1.5}Te_3$	V	240	410	12 mm	Ref.10
$Bi_2Te_{2.7}Se_{0.3}$	/	327	650	/	Ref.11
Bulk Te	V	400	550	1.5 mm	Ref.12
Te flake	H		180	5 μm	Ref.13
Te flake	H	413	180	50 μm	Ref.14
Te	V/H	647	Ambient temperature	~ 100 nm	This work

Supplementary Movie S1. Appearance demonstration of the T-scope sensor.

Supplementary Movie S2. The perceived 3D force vs. ground-truth 3D force testing.

Supplementary Movie S3. Visual-tactile endoscopy experiment of human simulated organ (Bronchus).

Supplementary Movie S4. Visual-tactile endoscopy experiment of human simulated organ (Intestine).

Supplementary Movie S5. Visual-tactile endoscopy experiment of human simulated organ (Stomach).

Supplementary Movie S6. In-vivo animal visual-tactile diagnosis validation experiment.

Supplementary References

1. Jin, M. *et al.* Visual tactile sensor based on infrared controllable variable stiffness structure. *IEEE Sens. J.* **21**, 27076–27083 (2021).

2. Zhang, C. *et al.* Learning-based six-axis force/torque estimation using gelstereo fingertip visuotactile sensing. in *Proc. IEEE/RSJ International Conference on Intelligent Robots and Systems (IROS)* 3651–3658 (IEEE, 2022).

3. Xu, R. *et al.* Deep flow-guided video inpainting. in *Proc. IEEE/CVF Conference on Computer Vision and Pattern Recognition* 3723–3732 (IEEE, 2019).

4. Zhou, S. *et al.* Propainter: Improving propagation and transformer for video inpainting. in *Proc. IEEE/CVF International Conference on Computer Vision* 10477–10486 (IEEE, 2023).

5. Zhang, K. *et al.* Inertia-guided flow completion and style fusion for video inpainting. in *Proc. IEEE/CVF Conference on Computer Vision and Pattern Recognition* 5982–5991 (IEEE, 2022).

6. Zhang, K. *et al.* Flow-guided transformer for video inpainting. in *European Conference on Computer Vision* 74–90 (Springer, 2022).

7. Zhou, M. *et al.* Ultrahigh thermoelectricity obtained in classical BiSbTe alloy processed under super-gravity. *Nat. Commun.* **16**, 7645 (2025).

8. Gong, T. *et al.* High-performance planar thin-film thermoelectric cooler based on sputtered nanocrystalline Bi2Te3/Bi0.5Sb1.5Te3 thin films for on-chip cooling. *ACS Appl. Mater. Interfaces* **17**, 17008–17017 (2025).

9. Zheng, G. *et al.* High thermoelectric performance of p-BiSbTe compounds prepared by ultra-fast thermally induced reaction. *Energy Environ. Sci.* **10**, 2638–2652 (2017).

10. Hao, F. *et al.* High efficiency Bi2Te3-based materials and devices for thermoelectric power generation between 100 and 300 °C. *Energy Environ. Sci.* **9**, 3120–3127 (2016).

11. Kim, J. H. *et al.* Possible Rashba band splitting and thermoelectric properties in CuI-doped Bi2Te2.7Se0.3 bulk crystals. *J. Alloys Compd.* **806**, 636–642 (2019).

12. Lin, S. *et al.* Tellurium as a high-performance elemental thermoelectric. *Nat. Commun.* **7**, 10287 (2016).

13. Wu, X. *et al.* Unprecedently low thermal conductivity of unique tellurium nanoribbons. *Nano Res.* **14**, 4725–4731 (2021).

14. Qiu, G. *et al.* Thermoelectric performance of 2D tellurium with accumulation contacts. *Nano Lett.* **19**, 1955–1962 (2019).
