# Supplementary material for: Superelastic Tellurium Thermoelectric Coatings for Advanced Trimodal Microsensing
Source: Nat Commun. 2026 Jan 13;17:1612. doi: 10.1038/s41467-026-68317-3 (PMC12905337; doi:10.1038/s41467-026-68317-3)
Supplement: Supplementary file 2 — Description of Additional Supplementary Files [file 41467_2026_68317_MOESM2_ESM.pdf]

Description of Additional Supplementary Files:

Supplementary Movie S1. Appearance demonstration of the T-scope sensor.

Supplementary Movie S2. The perceived 3D force vs. ground-truth 3D force testing.

Supplementary Movie S3. Visual-tactile endoscopy experiment of human simulated organ (Bronchus).

Supplementary Movie S4. Visual-tactile endoscopy experiment of human simulated organ (Intestine).

Supplementary Movie S5. Visual-tactile endoscopy experiment of human simulated organ (Stomach).

Supplementary Movie S6. In-vivo animal visual-tactile diagnosis validation experiment.
